# Human dignity and the creation of human–nonhuman chimeras

**DOI:** 10.1007/s11019-015-9644-7

**Published:** 2015-05-16

**Authors:** César Palacios-González

**Affiliations:** Institute for Science Ethics and Innovation, The University of Manchester, Oxford Road, Stopford Building, Room 3.383, Manchester, M13 9PL UK

**Keywords:** Chimeras, Human–nonhuman chimeras, Human dignity, Dignity, Nonhuman animals, Part-human

## Abstract

In this work I present a detailed critique of the dignity-related arguments that have been advanced against the creation of human–nonhuman chimeras that could possess human-like mental capacities. My main claim is that the arguments so far advanced are incapable of grounding a principled objection against the creation of such creatures. I conclude that these arguments have one, or more, of the following problems: (a) they confuse the ethical assessment of the creation of chimeras with the ethical assessment of how such creatures would be treated in specific contexts (e.g. in the laboratory), (b) they misrepresent how a being could be treated solely as means towards others’ ends, (c) they fall short of demonstrating how humanity’s dignity would be violated by the creation of such entities, and (d) they fail to properly characterise the moral responsibilities that moral agents have towards other moral agents and sentient beings.

## Introduction

In this paper I present and critically examine the dignity-related arguments that have been advanced against the creation of human–nonhuman chimeras that could possess human-like mental capacities. The paper is divided into three main sections. In this first section I present a brief account of what chimeras are and what role they play in biological sciences research. In the second section I examine, and show the pitfalls of, the human dignity definitions that for the most part have been used when arguing against the creation of such chimeras. In the third section I investigate the dignity-related arguments advanced by Karpowicz et al. (2004, 2005), Johnston and Eliot (2003), de Melo-Martín (2008), and MacKellar and Jones (2012) and show why they are found wanting.

While Karpowicz et al.’s arguments have been examined before,^1^ this paper adds to the current discussion on the ethics of creating human–nonhuman chimeras in several new ways. First, I present new counterarguments against Karpowicz et al.’s position. Second, I explore, for the first time, the arguments advanced by Johnston and Eliot, de Melo-Martín, and Mackellar and Jones. Finally, I show that from a species neutral perspective the dignity-related arguments that have been advanced against the creation of chimeras with human-like mental capacities do not only apply to the creation of human–nonhuman chimeras that are *preponderantly nonhuman*, but also apply to human–human chimeras, and to human–nonhuman chimeras that are predominantly human.

### The mythological chimera

We owe the canonical characterisation of the mythological Chimera to the Ancient Greek epic poet Homer. In the sixth book of the Iliad (179–181) Homer narrates how Glaucus, captain in the Lycian army, is going to face the Greek hero Diomed in single combat. Prior to their fight, Diomed asks who is he to face for fear that his opponent might be a god. Glaucus responds by telling the story of his lineage, and reveals that he is the grandson of Bellerophon, who by command of King Iobates killed Chimera. It is in these lines of the Iliad that Homer depicts this mythological creature:

First, dire Chimaera’s conquest was enjoin’d;

A mingled monster of no mortal kind!

Behind, a dragon’s fiery tail was spread;

A goat’s rough body bore a lion’s head;

Her pitchy nostrils flaky flames expire;

Her gaping throat emits infernal fire. (Homer 1836)

Homer’s characterisation is not the only one to be found in Ancient Greek mythology. In the Theogony (319–325), Hesiod describes Chimera not as a one-headed being with the body parts of three different animals, but as a creature composed of the heads and body parts of three different animals:

She [Echidna] was the mother of Chimaera who breathed raging fire, a creature fearful, great, swift footed and strong, who had three heads, one of a grim-eyed lion, another of a goat, and another of a snake, a fierce dragon; in her forepart she was a lion; in her hinder part, a dragon; and in her middle, a goat, breathing forth a fearful blast of blazing fire. (Hesiod 1914)

In the broader mythological context the term chimera has come to mean any entity that is constituted of different parts of different kinds of animals. For example, sirens, harpies, centaurs and the Minotaur are considered to be chimeric entities (Anijar and Brem 2003). Chimeras, in this wider sense, are depicted in Ancient Greek mythology both as dangerous creatures—like the sirens, who succeeded at shipwrecking sailors, and the Minotaur, who devoured those sent into his labyrinth—and as noble creatures—like the centaur Chiron, who trained Heracles.

### Non-mythological chimeras

In the second half of the twentieth century, interspecific chimeras ‘escaped’ the bounds of mythology and literary studies to become intentionally created and studied in life sciences faculties around the world.^2^ Chimeras, contrary to transgenic animals, are not created by the insertion of one, or multiple, exogenous genes. They are beings formed by combining the whole cells of genetically different organisms into a single organism.^3^ The UK Academy of Medical Sciences provides this definition, which I will endorse throughout the paper:

Chimæras are formed by mixing together whole cells originating from different organisms. The new organism that results is made up of a “patchwork” of cells from the two different sources. Each cell of a chimæra contains genes from only one of the organisms from which it is made. (…) Primary chimæras are formed by mixing together two early embryos, or an early embryo with isolated embryonic cell types obtained from a different embryo or cultured stem cell line. The resulting chimæra has cells of different origins, in many tissues. Secondary chimæras are formed experimentally by transplanting (or grafting) cells or tissues into animals at later stages of development, including late fetal stages, post-natal or even adult animals. The donor cells are only present in a few tissues. (The Academy of Medical Sciences 2011, 18–19)

Two of the first chimeras to be intentionally created were a goat-sheep chimera and a quail-chick chimera (Fehilly et al. 1984; Le Douarin et al. 1974; Balaban et al. 1988). Contrary to these *intentionally* created chimeras they can also occur naturally, for example when two non-monozygotic early human embryos fuse inside the womb (Tippett 1983; Norton and Zehner 2008).

Intentionally created chimeras can be intraspecific—when the cells that create such creatures originate within organisms (or cultured stem cell lines) that belong to the same biological species—or they can be interspecific—when the cells that create such beings originate within organisms (or cultured stem cell lines) that belong to different biological species (Xiang et al. 2008). It is worth noting, as the definition of the Academy of Medical Sciences points out, that the production of chimeras is affected by the number of engrafted cells, the origin of those cells, and by the time of the mixing. This means that alterations in these variables would produce different outcomes in respect of the kinds of beings produced. Having this in mind should guard us against what John Harris calls the ‘mermaid myth’: the idea that if we create a chimeric entity the resulting creature would necessarily possess easily recognizable phenotypic features from all ‘progenitors’ (Harris 2011).

Along with chimeras and transgenic animals there is another type of being that can be created both naturally and through biotechnology: hybrids. Hybrids are the offspring of organisms that belong to different biological species. For example, a mule is a hybrid produced by a female horse and a male donkey. Humans can ‘produce’ hybrids in two ways: through artificial reproductive techniques (artificial insemination or in vitro fertilization) or by setting the conditions so a fertile male and female of different biological species mate. The viability of the hybrids, in both cases, will depend on biological factors.

### Human–nonhuman chimeras

Chimeras, primary and secondary, can be produced with human components. In biological sciences human–nonhuman chimeras (henceforth HNH-chimeras) have been used in research into human haematopoiesis, the development and function of the immune system, infectious diseases, autoimmunity, cancer, and regenerative medicine (Shultz et al. 2007). They have also been used as research tools for the creation of vaccines against deadly diseases such as malaria, dengue, Hepatitis B, HIV and Hepatitis C (Davis and Stanley 2003; Sacci Jr et al. 2006; Yauch and Shresta 2008; Legrand et al. 2009; Bhan et al. 2010); and have been employed for the study of human cell development, maturation and migration (Sun et al. 2007; Tam and Rossant 2003; Lapidot 2001).

The creation and use of most contemporary human-developed HNH-chimeras has not been seen to present new ethical concerns, other than those related to the destruction of human embryos, animal ethics and research ethics. In part, this has been the case because such entities have been predominantly constituted by nonhuman components. Nonetheless, the possibility of creating HNH-chimeras with brains composed largely of human brain cells has raised ethical concerns about the morality of such experiments (Greely et al. 2007). The main question is if it is morally permissible to create HNH-chimeras that would possess the capacities that are generally associated with ‘human’ dignity or personhood.^4^ For example, is it morally permissible to engraft sufficient human stem cells, or neural stem cells, into a great ape embryo so it develops normal human-like cognitive capacities?

In response to the possibility of creating HNH-chimeras with dignity, or personhood, related capacities, several authors have advanced dignity-related arguments in order to prove that doing so *would be immoral and should not be done*. Now, before examining such arguments (see Sect. ‘Dignity and HNH-chimeras with human-like mental capacities’) I will commence by emphasising a well-known problem with dignity-related stances: that there is no consensus about what dignity means (the term has even been regarded as a useless concept; Schroeder 2010; Macklin 2003). Having this in mind, in the next section I will not try to provide a final account of dignity, but I will critically examine the definitions that Karpowicz et al. (2004, 2005) have proposed.

## ‘Human’ dignity

Karpowicz et al. have argued that even when the creation of certain types of HNH-chimera is morally *unproblematic*, human dignity would be denied, undermined or denigrated by the creation of HNH-chimeras that possess human-like functional and emergent psychological capacities^5^^,^^6^ (Karpowicz et al. 2004, 2005). Let’s examine what they understand by human dignity.

In their 2004 paper Karpowicz et al. defined human dignity as a “widely shared concept that refers to being worthy or respected because one is human” (Karpowicz et al. 2004, 333). According to this definition, humans’ moral value is grounded on their belonging to the *Homo sapiens* species (according to the common understanding of what ‘human’ means). Thus, any being that belongs to this biological species would possess human dignity, and any being that does not belong to it would not possess human dignity.

Karpowicz et al. go on to clarify that “Human dignity is based on the recognition that human beings possess, will possess, or have possessed functional and emergent psychological capacities that indicate they are worthy of respect” (Karpowicz et al. 2004, 333). We can assert that Karpowicz et al.’s definition of human dignity entails the following elements:

1. Human dignity refers to being worthy or respected because one is human.
2. Human dignity is based on the recognition that humans possess, will possess or have possessed functional and emergent psychological capacities that indicate they are worthy of respect.

There are two things that must be noted about this definition. First, that it does not explicitly state the sort of moral status beings with dignity possess. Let’s remember that to state that an entity possesses moral status is to realise that “*in its own right and for its own sake, it can give us reason to do things such as not destroy it or help it*” (Kamm 2007, 229). Even so, the authors implicitly assume, as it will become clear, that to possess dignity is to have a unique moral value.

Second, there is a tension between their two clauses. The source of this tension is that 1 is far more extensive than 2. For 1, being human is a necessary and sufficient condition for possessing human dignity, while for 2 being human is a necessary but not a sufficient condition for possessing human dignity. While it is embedded in both clauses that being human is a necessary condition for possessing dignity, the sufficiency requirements are different in each case.

Why is this important? It is so because on the one hand, on 1, every human being—from the moment of conception to the moment of death—possesses human dignity. For example, an anencephalic child possesses human dignity. While, on the other hand, on 2, there are some human beings that do not fulfil the conditions for possessing human dignity. Human beings that do not possess, will not possess and have never possessed functional and emergent psychological capacities cannot be recognized as possessing human dignity. For example, a child with genetically caused anencephalia. The question that Karpowicz et al. have to answer is how anencephalic child type cases fit into their account of human dignity without being inconsistent with a definition that includes both 1 and 2.

Baylis and Fenton think that the only way Karpowicz et al. can make their human dignity definition work is by endorsing the idea that belonging to a class that contains members that possess certain cognitive or emotional capacities (thus effectively renouncing to 2) is what grants such moral worth:

At the same time, both of these points in tension [what I have called *1* and *2*] rely on an implicit appeal to a principle conferring intrinsic moral value on *x* if *x* belongs to a class *A* that contains members who manifest certain cognitive or emotional capacities, even if *x* herself does not. *X* is thus valued, or possesses moral significance, because *x* is a member of class *A*. In this case, the class is all humans. (Baylis and Fenton 2007, 201)

If this is the case then a better way of expressing Karpowicz et al.’s human dignity definition would be:

1. Human dignity refers to being worthy or respected because one is human.
2. Human dignity is based on the recognition of being worthy or respected because one belongs to the *Homo sapiens* species, which is characterised by the fact that normally humans possess, will possess or have possessed functional and emergent psychological capacities.

There are at least two problems with this ‘new’ approach to human dignity. The first is that even when it clearly explains who possesses human dignity it does not explain why belonging to such a class (i.e. the *Homo sapiens* species) confers such moral worth—this objection has been long noted in animal ethics literature. It is simply stated, but not explained, that any member of the class *humans* has dignity. Now, if they maintain that species belonging *is what* confers such unique value then this definition is speciesist. Speciesism, in this case anthropocentrism, asserts that our human biological commonality confers us superior moral worth than those who are not members of our species (Singer 2009). Just as with racism and sexism, speciesism extracts a normative conclusion (humans have more moral worth than all other creatures) from an arbitrarily chosen morally insignificant fact. To be a member of the *Homo sapiens* species “is simply a matter of biology: It is to be an organism that has descended from a particular branch of the tree of terrestrial life, an organism whose genome lies somewhere within a particular range, or the like” (DeGrazia 2007, 312). Finally, species belonging appears not to be what confers dignity. For example, if we were to find extraterrestrial life forms with mental capacities like ours we would most certainly accept that they possess dignity. For example, if in real life we found a Vulcan alien—Spock—we would accept that killing him is tantamount to killing a human person.

The second problem that arises from this account is that if we concede that being human (i.e. belonging to the *Homo sapiens* species) is a *necessary* condition for possessing *human* dignity, then using such a definition to construct *a generalargument* against the creation of HNH-chimeras with human-like mental capacities becomes deeply problematic. Why? Because certain HNH-chimeras with human-like mental capacities cannot be categorised as being human, and therefore they would not fulfil a necessary condition for possessing dignity: *belonging to the Homo sapiens species*. For example, a human-chimp chimera that is completely chimpanzee except for its human brain would certainly not classify as belonging to the *Homo sapiens* species. If we accept this, then we also have to accept that human dignity arguments cannot be raised against the creation of HNH-chimeras with human-like mental capacities in *all cases*. Therefore, even if Karpowicz et al.’s arguments were correct, the creation of certain HNH-chimeras with human-like mental capacities would not violate human dignity. Resnik (2003, 35) has made a similar point when commenting on the possibility of patenting a human embryo with chimpanzee genes: “I argued that it would threaten but not violate human dignity because the humanzee would not be a human being”.

One could challenge the previous point by arguing that HNH-chimeras belong to the *Homo sapiens* species by virtue of the engrafted cells, and thus have human dignity. A problem with this strategy is that those defending it would have to provide a reasonable explanation for how this happens and where the limits lie (i.e. how and under what circumstances the engraftment of *X* number cells ‘makes’ a nonhuman animal belong to the *Homo sapiens* species).^7^ They would also have to explain whether ‘species transition’ is bidirectional, or not, in cases concerning human beings (i.e. if we engraft *X* amount of nonhuman-animal cells into a human would such a human ‘become’ part of that nonhuman animal species). As Streiffer (2005, 357) asks, “when faced with an organism that has some human cells and some nonhuman cells, how is one to decide whether the organism is human, and hence, whether it is a human being?”.

The tension between clauses 1 and 2 can be solved by appealing to an anthropocentric principle (i.e. reformulating 2 into 2′). However, the cost of maintaining such a definition of human dignity is that we are stuck with a speciesist account that cannot support a general dignity-based argument against the creation of HNH-chimeras with human-like mental capacities. This being the case, we need to look for an alternative human dignity account if we want to advance a general argument against the creation of such creatures.

### A psychological capacities account of ‘human’ dignity

There is another way in which to interpret Karpowicz et al.’s human dignity definition, so that it could support a general critique of the creation of HNH-chimeras with human-like mental capacities. This interpretation holds that what grants dignity is the possession of certain psychological capacities. In this instance, we need to reformulate 1:

1. Human dignity refers to being worthy or respected because one is a human that possesses, will possess, or has possessed functional and emergent psychological capacities.
2. Human dignity is based on the recognition that humans possess, will possess or have possessed functional and emergent psychological capacities that indicate they are worthy of respect.

When we reformulate 1 we admit that there are some humans that do not, and cannot, possess human dignity—all those human beings that do not possess, will not possess and have never possessed functional and emergent psychological capacities. It becomes clear that Karpowicz et al. are embracing a potentiality account of moral worth,^8^ and thus the number of humans that do not possess dignity are reduced to those that by means of their congenital constitution lack the potentiality to develop such psychological capacities. At this point, let’s specify the mental capacities that they regard as confering moral worth.

According to Karpowicz et al., human beings’ moral worth does *not only* depend on the capacities of reasoning, choosing freely, and acting for moral reasons [as Kant (1998) proposes], or entertaining and acting on the basis of self-chosen purposes [as Gewirth (1982) asserts]. Karpowicz et al. (2005, 120) instead assert that what grants certain humans their unique worth are the previous capacities plus “those for engaging in sophisticated forms of communication and language, participating in interweaving social relations, developing a secular or religious world-view, and displaying sympathy and empathy in emotionally complex ways”. For them, dignity is a cluster concept where none of the former capacities by itself is sufficient for possessing dignity, but when they appear together they paradigmatically point towards what it is to have dignity.

Now, if we accept that species belonging is a morally insignificant fact, then we have to accept that species membership in 1′ and 2 should also be regarded as morally irrelevant. This being the case, we can actually remove this condition (namely, belonging to the *Homo sapiens* species) without any loss. In fact, the definition of human dignity that these authors provide in their 2005 paper could be interpreted as pointing in that direction: “Human dignity is a widely shared notion that signifies that humans typically display certain sorts of functional and emergent capacities that render them uniquely valuable and worthy of respect” (Karpowicz et al. 2005, 120).

It is important to note that this definition is not a direct quote from their 2004 paper, but a new definition that abandons 1, modifies 2 and specifies the value that human beings have. In fact, being human is eliminated as *a necessary condition* for possessing dignity, and thus leaves open the possibility for other beings to possess dignity. If a capacities-based interpretation of dignity is warranted then their assertion that “The family of capacities associated with human dignity seems to belong uniquely to human beings” (Karpowicz et al. 2005, 122) could in fact be construed as claiming that *so far* there is no other being (biological or non-biological) known to humans that possesses such capacities.^9^ Their second definition could be stated, in a species neutral fashion, as follows:

(3) Dignity is a widely shared notion that signifies that some beings typically display certain sorts of functional and emergent capacities that render them uniquely valuable and worthy of respect.

Karpowicz et al. realise that if they adopt a capacities account of dignity then there are humans that could not be viewed as possessing it. Confronted with this scenario they embrace Alan Gewirth’s position when dealing with so called ‘marginal cases’.^10^ For Gewirth (1982, 27–28) dignity is “a characteristic that belongs permanently and inherently to every human as such”. A problem with this strategy is that Gewirth’s stance is *inconsistent* with a logical implication of Karpowicz et al.’s capacities based approach—namely, that there are humans that do not possess dignity. The authors try to solve this inconsistency in the following way:

We tend to ascribe it [dignity] to all humans, no matter how seriously impaired or ill they may be, because there is no clear agreement about just how many dignity-associated capacities a person must possess to be said to have human dignity. To avoid the possibility of mistakenly failing to treat those with severe disabilities as ends in themselves, human dignity proponents ascribe dignity to all humans. (Karpowicz et al. 2005, 121–122)

This solution can be formulated as:

(4) We ascribe dignity to *all humans* because there is no agreed amount of dignity-related capacities one must possess in order to have dignity, and also to avoid the mistake of treating seriously impaired or ill humans as mere means rather than as ends in themselves.

Robert Streiffer has argued that such a solution is not warranted because there are clear cut cases where a human being does not possess such morally worthy capacities:

[A]n appeal to uncertainty and disagreement seems implausible given that there is no real uncertainty or disagreement that a newborn fails to have the capacities they cite and so would, on their view, clearly lack the special moral status that accompanies individuals with human dignity. (Streiffer 2005, 357)

While I agree with Streiffer that there are clear cut cases that do warrant such a solution, it must be said that Karpowicz et al. could claim that there is a problem with his counterexample: because they assume a potentiality account, Streiffer’s new born counterexample does not work in all cases. It does not work because a new born possesses dignity in so far as she possesses the dignity-related capacities in a potential state.^11^

Karpowicz et al. are correct that there are cases where a ‘prudential’ solution is warranted (e.g. where the amount of capacities possessed by a being situates her in a grey area), but there are other cases where it is clearly not warranted (e.g. when humans, due to a congenital condition, do not possess the potential to develop such capacities). Given that there are cases where this solution is unwarranted (e.g. anencephalic cases), Karpowicz et al. should abandon it in its present form. If they do not then they will have to accept that they are proposing an *ad hoc* speciesist solution.

Karpowicz et al.’s dignity account can be interpreted in two ways. The first way has an explanatory gap, and cannot ground a general argument against the creation of HNH-chimeras with human-like mental capacities. We should therefore abandon it. The second interpretation, because it focuses on capacities and is species neutral, can be used to construct a general argument against the creation of such chimeras. Throughout the rest of the paper I will adhere to the second interpretation, although I will leave out Karpowicz et al.’s Gewirth-like solution to the species-overlapping cases given that it is not warranted. Now I will assess the dignity-related arguments that have been presented against the creation of HNH-chimeras with human-like mental capacities.

## Dignity and HNH-chimeras with human-like mental capacities

(I) The first argument that Karpowicz et al. (2004, 333) propose against the creation of HNH-chimeras is that “Chimeras, by combining the appearance and functional capacities of humans and animals, seem to risk denying human dignity”. The authors assert that the first part of this argument should be dismissed, because dignity has nothing to do with the outward appearance of HNH-chimeras or with the intuitions that such appearances might elicit from us. The argument, after removing its redundant section, could be expressed like this:

(5) HNH-chimeras risk denying human dignity by combining the functional capacities of humans and nonhuman-animals.

However, we should also eliminate ‘human’, from ‘human dignity’, so to favour a species neutral dignity argument:

(5′) Chimeras risk denying dignity by combining the functional capacities of humans and nonhuman animals.

According to this argument—which is similar to that advanced by the US National Academy of Science (National Academies of Science 2005, 55)—the creation of HNH-chimeras whose psychological capacities are a ‘combination’ of human and nonhuman ones risks denying dignity. The first problem with this argument is that it is not clear *which HNH*-*chimeras* risk denying dignity. If it indicates that *all HNH*-*chimeras*—whose psychological capacities are a combination of human and nonhuman mental capacities—risk denying dignity (as it seems to imply), then it is easy to provide a thought experiment that calls this argument into question. Suppose that a human person gives her informed consent for her brain to be grafted with modified elephant neural stem cells, in order to treat a memory disorder. This action creates a HNH-chimera that ‘combines’ the functional capacities of humans and nonhuman-animals. Even so it does not appear to risk denying dignity, and if it does it is not clear why.

If the authors are instead suggesting that the creation of ‘predominantly’ nonhuman HNH-chimeras—where the nonhuman animal component belongs to a species that does not possess human-like psychological capacities—is what risks denying dignity then they face another problem: when we create a HNH-chimera that effectively combines human and nonhuman functional capacities we do not deny dignity but rather *a being with dignity is created*. For example, if we could engraft enough human neural stem cells into a pig embryo that it develops human-like mental capacities, then we would have created a HNH-chimera with dignity. The creation of this HNH-chimera does not deny dignity, as before chimerisation the pig embryo does not possess dignity to be denied.^12^

(II) The second argument that Karpowicz et al. advance states that ‘human’ dignity would be undermined by the transfer of emergent psychological human functions into *research subjects*. They claim:

If such a chimera exhibited signs of emergent human mental capacities, conducting biomedical experiments upon it might be essentially equivalent to conducting the same experiments on a human person. Human dignity would be undermined by the transfer of emergent and supercellular psychological human functions into research subjects that by consequence would possess the same capacities themselves. (Karpowicz et al. 2004, 333–334)

The argument encompasses two elements and can be expressed as follows:

(6) If a chimera exhibited signs of emergent human mental capacities, conducting biomedical experiments upon her might be essentially equivalent to conducting the same experiments on a human that possessed dignity.
(7) Dignity would be undermined by the transfer of emergent and supercellular psychological human functions into research subjects that by consequence would possess the same capacities themselves.

It is true that if a HNH-chimera possessed the same mental capacities as a human person, then we should assess the morality of the procedures carried out upon her as if they were carried out upon a human person. But it is false that dignity would be undermined if the recipient of such capacities was a research subject that by virtue of the procedure had gained these capacities. If Karpowicz et al.’s argument is correct then the following case would also undermine dignity: suppose that scientists engraft normal human stem cells into a pre-term congenitally anencephalic child. Imagine that by virtue of this intraspecific chimerisation process this research subject is able to develop, otherwise unattainable for her, normal human mental capacities. Now, it is evident that this procedure does not undermine dignity by means of transferring emergent and supercellular psychological human functions *into a research subject* (in this case a human research subject).

Karpowicz et al.’s argument could also be interpreted as stating that dignity is undermined when dignity-possessing research subjects are not treated as possessors of dignity, a claim which is endorsed by de Melo-Martín (2008, 338) and Streiffer (2005, 362–366). It should be noted that if this is the true sentiment of the argument, then it cannot be advanced as a principled objection against the creation of HNH-chimeras with human-like mental capacities. Why not? Because such interpretation *disaggregates* the ethics of creating HNH-chimeras with human-like mental capacities from the ethical evaluation of how research subjects are treated. At best such an argument would reveal something that everybody accepts, namely that moral agents should treat other beings according to their moral status. Greene et al. (2005, 386) have advanced a similar point in asserting that one option is to not create HNH-chimeras, and the other option is “to understand the mental capacities of engrafted animals and to treat them in a manner appropriate to their moral status”. At this point we can reformulate 7 into 7′:

(7′) Dignity cannot be undermined by the transfer of emergent and supercellular psychological human functions into research subjects that by consequence would possess the same capacities as human persons, but it can be undermined by the mistreatment that such subjects might receive from other moral agents.

(III) Karpowicz et al.’s third argument focuses on the impact that creating HNH-chimeras with human-like mental capacities would have on the possibility that humans could exercise their own dignity-related capacities:

[A]n argument from human dignity would maintain that to create a human-nonhuman chimera would either diminish or wholly eliminate the possibility that humans could exercise the cluster of capacities and characteristics that are associated with human dignity, treating them solely as a means to others’ ends. (Karpowicz et al. 2005, 121)

To state that the mere creation of a being with dignity diminishes or eliminates the possibility that other humans (or other beings with dignity) could exercise their dignity-related capacities is mistaken. If this assertion was true then it would also be true that every time an extraterrestrial alien person is born, supposing that there are human-like intelligent aliens in the universe, her birth would somehow diminish or eliminate the possibility that humans could exercise their dignity-related capacities. To assume that every alien person’s birth leaves all human persons worse off in this sense is clearly false. The fact that another being with dignity is created does not affect humans’ ability to exercise their dignity-related capacities. DeGrazia (2007, 236), along these same lines, has rightly pointed out that if we coexisted with other hominid non *Homo sapiens* borderline, or paradigmatic, persons, their existence would not diminish or eliminate the possibility that we could exercise our dignity-related capacities. He provides the following example: if we were to find a living member of the *Homo floresiensis* species this would not diminish or eliminate the possibility that we, humans, could exercise our dignity-related capacities.

A more charitable interpretation of this argument could be offered: we could assume that Karpowicz et al. are not talking about *humans* (in “humans could exercise”) but about *HNH*-*chimeras*. Even so, the argument remains problematic. First let’s see a reconstructed version of it:

(8) An argument from dignity would maintain that to create a human–nonhuman chimera would either diminish or wholly eliminate the possibility that human–nonhuman chimeras could exercise the cluster of capacities and characteristics that are associated with dignity, treating them solely as a means to others’ ends.

This revised version of the argument is problematic in two ways. The first problem mirrors that of the ‘uncharitable’ interpretation of it. How could the creation of HNH-chimeras with dignity-related capacities by itself cause that other HNH-chimeras could not exercise their dignity-related capacities? This seems plainly false. Second, even if the argument is to be understood as stating that to create a HNH-chimera would either diminish or wholly eliminate the possibility that she could exercise her dignity-related capacities, given that she will be treated solely as a means to others’ ends, it remains problematic. First, it does not *necessarily* follow from the fact that HNH-chimeras with dignity-associated capacities are created that they would be treated merely as means towards others’ ends. Here it is implied, incorrectly, that researchers would be oblivious to, or negligent of, the HNH-chimeras’ moral status. Secondly, it is possible, as observed by most commentators, that researchers could overlook the chimera’s moral status but it does not follow from this possibility that creating HNH-chimeras with human-like mental capacities would violate dignity. If this was true then it would also be true that slaves violate dignity when they intentionally have children that in turn will be slaves, given that such children are going to be treated solely as means to others’ ends, and this is clearly false.

(IV) The fourth argument that Karpowicz et al. advance is grounded on the degree of functionality of the dignity-related capacities that HNH-chimeras would possess:

By giving nonhumans some of the physical components necessary for development of the capacities associated with human dignity, and encasing these components in a nonhuman body where they would either not be able to function at all or function only to a highly diminished degree, those who would create human-nonhuman chimeras would denigrate human dignity. (Karpowicz et al. 2005, 121)

There are three problems with this line of argumentation. First, from a species neutral version of it, the implausible conclusion that we should not ‘give’ these necessary physical components (e.g. neural tissues) to certain humans that are congenitally severely cognitively impaired would follow. According to the argument, we should not give such physical components to those congenitally severely cognitively impaired humans that have bodies where the components would either not be able to function at all or function only to a highly diminished degree. This strikes us as evidently false.

Secondly, given that Karpowicz et al. endorse a functionality threshold for what counts as a denigration of dignity it would also follow—if we rejected their potentiality account—that we denigrate human dignity when we restore someone’s dignity-related capacities to a highly diminished degree. This is an implausible conclusion: we can easily imagine a case where at time T^1^ someone possesses all dignity-related capacities, then at T^2^ she loses them all due to an accident or illness, and then at T^3^ some of the physical components necessary for the development of the dignity-related capacities are restored by a doctor through an intraspecific or interspecific chimerisation process. The only caveat is that at T^3^ the physical components necessary for the development of such dignity-related capacities are encased in a body where they would not be able to function at all or they would only function to a highly diminished degree. Karpowicz et al. would have to accept that these ‘restorative’ procedures would denigrate dignity. This, again, strikes us as false.

The third, and final, problem with this argument is that it incorrectly assumes that such procedures would *diminish or eliminate the capacities associated with dignity*, when encasing the physical components necessary for their development in a body where they would either not be able to function at all or function only to a highly diminished degree. “The creator of the human–nonhuman chimera would do even worse [than a torturer or enslaver]—he or she knowingly would diminish or eliminate the very capacities associated with human dignity” (Karpowicz et al. 2005, 121). Now, this is incorrect because prior to the procedure there are no dignity-related capacities, at least not those that Karpowicz et al. specify, that could be *diminished or eliminated.* As de Melo-Martín (2008, 342) points out, “such capacities cannot be destroyed or diminished unless there already is a creature with those capacities full present”. Contrary to what Karpowicz et al. state, a certain degree of dignity-related capacities would emerge, but such emergence is dependent on other biological factors (e.g. the body where the human neural stem cells are transplanted).

(V) The fifth, and final, argument that Karpowicz et al. advance is a variation of the ‘treatment-argument’ presented in their 2004 paper. In this new version, they state that to create a HNH-chimera with dignity-related capacities would denigrate dignity because the HNH-chimera would not be able to exercise such capacities due to its role as a research subject:

To create such a chimera would violate human dignity because the resulting being could not fully exercise the dignity-related capacities associated with the human brain, due to its role as a research subject specifically produced to serve as a human proxy in experiments that it would be unethical to undertake on human beings themselves. (Karpowicz et al. 2005, 123)

This argument is problematic because, as explained previously, such treatment of a HNH-chimera is not a *necessary* feature of its creation. It does not necessarily follow from the fact that someone is an experimental subject that she will not be able to exercise their dignity-related capacities. Secondly, irrespective of the intentions for which the HNH-chimeras were created, moral agents have a *moral obligation* to treat them in accordance with their moral status. Just as it would be immoral for a researcher to carry out harmful or destructive experiments on a child created for the purpose of those experiments, it would be immoral to fail to respect the moral value that HNH-chimeras possess by means of their capacities.

So far I have tried to show that Karpowicz et al.’s dignity-related arguments against the creation of specific HNH-chimeras—those with functional and emergent psychological capacities—fail to prove that *in principle* their creation would violate, deny or denigrate dignity. Now I will turn to examine three arguments that Johnston and Eliot have advanced against the creation of HNH-chimeras with human-like mental capacities. Before examining these authors’ arguments, it must be said that for them the only HNH-chimeras that risk offending dignity are those that are compromised (i.e. those that are harmed by virtue of being a mix of human and nonhuman).

(VI) Johnston and Eliot argue that dignity would be offended in so far as “[i]ntentionally creating compromised human beings or part-human beings is cruel to the creature created (…)”^13^ (Johnston and Eliot 2003, W7). Along the same lines, MacKellar and Jones have argued that “Indeed, it seems that the attempt to create a part human, part nonhuman being would be wrong to that being” (MacKellar and Jones 2012, 176).

I should note that this argument cannot be levelled as a *general argument* against the creation of HNH-chimeras with human-like mental capacities. Why? Because in *creation contexts* it is important to take into consideration the non-identity problem as identified by Parfit (1984). The non-identity problem may be interpreted to show that reproductive choices, or in this case the creation of some HNH-chimeras, cannot harm the created individual unless her life is a life not worth living.^14^ This is so under a comparative account of harm, and because her only other ‘option’ was never to have been. Cooley (2008, 2) has made a similar point: “As long as [HNH-chimeras] have lives worth living or good lives, one cannot say legitimately neither that their creation and existence injured them in some way nor that their existence are inherently bad”. This being the case, we must reject this argument as a general argument against the creation of HNH-chimeras with human-like mental capacities.

(VII) The second argument that Johnston and Eliot advance maintains that the creation of such chimeras reflects badly on those creating them and those allowing their creation:

Intentionally creating compromised human beings or part-human beings reflects badly [and can be said to offend dignity] both on those who create the chimera and on those societies or governments allowing its creation. What kind of an institutional intention do we exhibit when we create compromised human beings or part-human beings for our laboratory use?” (Johnston and Eliot 2003, W7)

This argument cannot be weighed as a principled objection against the creation of compromised human beings or part-human beings (with our without human-like mental capacities). It cannot be so because the institutional intentions behind their creation are not *necessarily bad or evil*. Even more so, those intentions can be benevolent and on a par with treating such beings in accordance with their moral status. For example, the intention behind creating a ‘compromised’ human being, through a chimerisation procedure, could be to enhance the capacities of a congenitally severely cognitively disabled human. It is hard to see how this could offend dignity. Secondly, this argument, from a species neutral perspective, also entails that those who intentionally reproduce knowing that they may create a ‘compromised’ human (a severely ill or impaired human) would offend dignity and this (except in wrongful life cases) appears not to be the case.

(VIII) The third, and final, argument that Johnston and Eliot advance questions society’s role in determining the moral acceptability of the creation of HNH-chimeras with human-like mental capacities:

Finally, intentionally creating compromised human beings or part-human beings might appear to “all the world” to be using another human, or a part-human, as a means to an end rather than as an end in itself [and thus to offend dignity], a use that has been confirmed as morally unacceptable since at least the Declaration of Helsinki. (Johnston and Eliot 2003, W7)

This final argument is also unsound: the fact that something might appear to ‘all the world’ as X does not mean that it is morally on par with X and should not be done. Even when it might appear to ‘all the world’ that I am trying to drown a child when in fact I am trying to save her, it does not follow that I should stop trying to save her. Likewise, the fact that ‘all the world’ thinks that a HNH-chimera will be used solely as a means towards others’ ends does not mean that this is going to be the case. If we create HNH-chimeras with human-like mental capacities then we should treat them in accordance with their moral status. At this point it is safe to claim that Johnston and Eliot’s arguments are found wanting.

As well as advancing these arguments, Johnston and Eliot have pointed out that there are two ways of understanding human dignity arguments. The first one focuses on the dignity of individuals (i.e. the dignity of a human) while the second one focuses on the dignity of a class of individuals (i.e. humanity’s dignity). According to the authors the second approach may lay the ground for new criticisms of the creation of HNH-chimeras that possess human-like psychological capacities (Johnston and Eliot 2003). Alongside this idea, de Melo-Martín (2008) has advanced that previous critics of the dignity-related arguments—namely Françoise Baylis and Andrew Fenton, and David DeGrazia—have failed to properly identity if Karpowicz et al.’s arguments were directed at individuals or at a class of individuals. According to her, the class-based counterarguments advanced by Baylis and Fenton, and DeGrazia are skewed because Karpowicz et al.’s arguments concerned individuals:

[C]ritics and proponents of the human dignity argument do not have a similar understanding of how chimera research poses a threat to human dignity. Thus, although the critics’ arguments might be right, given that these arguments do not address the particular way in which proponents believe that human dignity would be threatened, they cannot conclude that this threat does not exist. (de Melo-Martín 2008, 343)

de Melo-Martín then advances two new arguments, from humanity’s stance, against the creation of HNH-chimeras with human-like mental capacities.

(IX) de Melo- Martín’s (2008) first argument states that a threat to humanity’s dignity would occur if scientists created HNH-chimeras with human-like mental capacities such that the chimeras were not able to flourish according to their nature. Even when this appears to be an individual dignity argument the author considers it otherwise. In order to show us why this is a humanity’s dignity argument she asks us to image a scenario where we replaced the HNH-chimeras for normal human beings:

It is clear, however, that were researchers to use human beings for experimental purposes, it would be reasonable to argue that such action would constitute a threat not just to the dignity of the particular humans involved, but also to the dignity of human beings as a whole. This would be the case, because all humans would be diminished by engaging in or condoning such activities. (de Melo-Martín 2008, 339)

This argument is problematic because it assumes that if all society knew about such practices, and it was clear that the experimental subject possessed dignity, all of society would engage in or condone such activities. Contrary to this assumption, I think that there would be substantial societal objection and that we would witness a large call to ban such research. While this is an empirical claim, I think that similar cases, for example people’s negative reactions to torture, the experimentation with great apes, and the hunting of dolphins, show that not all humans would engage in or condone such activities. Therefore, *not all humans* can be diminished by engaging in or condoning such activities because not all humans would engage in or condone such activities.

(X) The second argument that de Melo-Martín advances is that it is quite unlikely that HNH-chimeras will live in a context where society will allocate enough resources for them to flourish and function in accordance with their higher capacities:

[I]t is highly improbable that society would use resources to ensure that such creatures develop to the fullest extent of their capacities. Here again, the human dignity at stake would not be that of the creatures in particular, although their dignity might also be violated, but that of all human beings. (de Melo-Martín 2008, 339)

If this argument is correct then even without creating HNH-chimeras humanity’s dignity has be violated. Why? Because most human societies do not use their resources to ensure that all individuals within that society (abled and disabled) develop *to the fullest extent* their dignity-related capacities. On many occasions societies are not able to allocate such resources for reasons such as bad administration, because the resources available are scarce, or because they allocate resources to achieve other ends. For example, think of a hypothetical well-off society that decided to allocate most of its resources to fight climate change, and thus prevent an existential catastrophe. Because of this public schools’ funding is reduced and students are not able to develop their capacities to their *fullest extent*. If de Melo-Martín is correct then humanity’s dignity has been violated in this case, and this appears not to be the case.

A more charitable interpretation of this argument could be offered. It could be asserted that society should provide means for HNH-chimeras to be able to develop their dignity-related capacities to an *adequate extent*, otherwise humanity’s dignity would be violated. It is true that if we create HNH-chimeras with human-like mental capacities then we have a moral obligation to allocate the adequate resources so they develop to an adequate extent. It is also true that the amount of resources dedicated to this task would most probably depend on local or federal regulations. If this was the case then, just as in the previous argument, it seems more accurate to state that only the dignity of those that participated in or condoned the allocation of insufficient resources for the chimera to develop to an adequate extent would be diminished. In this case, we must assert that the dignity at stake is not that of all human beings.

(XI) The final argument that I will examine has been advanced by Calum MacKellar and David Jones. In their book *Chimera’s Children*, these authors posit that the intentional creation of ‘intermediate’ beings—those that undermine the biological distinctions between humans and nonhuman animals (e.g. chimeras or hybrids)—with an unclear moral status would be the first step in a slippery slope towards putting into question the dignity of all those that possess it:

[N]ew beings would begin to exist to whom/which it would be very difficult to ascertain, with any amount of certainty, whether or not universal, absolute, inalienable, and inherent dignity should be conferred. In addition, if a being were denied the inherent dignity to which he or she was entitled, then the dignity of every individual in the whole global network, including every human being, would be put into question. This is because the whole network of persons (whether or not they are 100 per cent human) would no longer be consistent, coherent or dependable. (MacKellar and Jones 2012, 196)

There is one fatal problem with this argument: it does not follow from the fact that someone’s dignity is denied (intentionally or accidentally) that the dignity of all dignity-possessing creatures would be put into question. For example, not even under the most abominable political regimes has the dignity of *all humans* been put into question. It is always the dignity of the slave, and not of the enslaver, that has been questioned. Throughout history enslavers have managed to construct consistent, coherent and dependable networks of exclusion without putting themselves into danger. It is true that some HNH-chimeras’ moral status could be uncertain but in such cases we should err on the side of caution when dealing with them, not for humanity’s sake but for their sake.

## Final remarks

In this paper I have tried to show that the dignity-based arguments that have been advanced so far fail to make a principled case against the creation of HNH-chimeras with human-like mental capacities. I engaged with arguments by Karpowicz et al. (2004, 2005), Johnston and Eliot (2003), de Melo-Martín (2008), and MacKellar and Jones (2012), and found all of them to be problematic. These arguments are problematic because: (1) they confuse the wrongness of creating HNH-chimera with the wrongs and harms that would be imposed upon such HNH-chimeras in certain contexts; (2) they misrepresent how a being could be treated solely as means towards others’ ends; (3) they do not provide a satisfactory account of how the creation of HNH-chimeras would violate humanity’s dignity; and (4) they disregard the fact that if such HNH-chimeras had dignity then moral agents would have the same moral obligations towards those chimeras as they do towards other beings with dignity (no matter that the HNH-chimeras were created with the intention of being research subjects).

## Footnotes

1 For previous critiques of Karpowicz et al.’s arguments see: Streiffer (2005), Robert (2006), Ravelingien et al. (2006), Baylis and Fenton (2007), DeGrazia (2007), de Melo-Martín (2008) and Cooley (2008).

2 It is worth mentioning that although contemporary chimeras are tamer than those depicted by the Ancient Greeks we cannot discard the possibility that someone could find a way to weaponise a chimeric being. (Scott 2006; Savulescu 2013).

3 For a discussion about the possible chimeric entities that could exist see Greely’s (2003) *Defining Chimeras… and Chimeric Concerns*.

4 Throughout the paper I will use the term ‘person’ to refer to beings that possess dignity conferring psychological capacities.

5 Unless specified otherwise when I talk about functional and emergent psychological capacities I am talking about statistically normal human-like functional and emergent psychological capacities.

6 Eberl and Ballard (2008, 45) have correctly asserted that it is largely unknown if the engraftment of undifferentiated pluripotent human stem cells into a nonhuman animal embryo could lead to the development of a functioning cerebrum supportive of self-conscious rational thought. Although, as Alter (2007) states, to imagine an ape-human viable hybrid or any other chimera that contains the best features of each may be to imagine science fiction I will proceed in this paper assuming that this might be possible. One case that partially supports this idea is the creation of mouse-human chimeras where their long-term potentiation “was sharply enhanced (…), as was their learning, as assessed by Barnes maze navigation, object-location memory, and both contextual and tone fear conditioning.” (Han et al. 2013, 342).

7 The creation of intraspecific chimeras does not present this indeterminacy problem.

8 There are different versions of the potentiality account. One of them is that human beings possess moral worth from the moment of conception because they have the potential to develop certain capacities that are not present during the first period of their development, that are morally worthy.

9 Karpowicz et al.’s qualifications for possessing dignity are so stringent (e.g. developing a secular or religious world-view) that no nonhuman animal appears to have them.

10 The argument from marginal cases, or argument from species overlap, points out that “the criteria that are commonly used to deprive nonhuman animals of moral consideration fail to draw a line between human beings and other sentient animals, since there are also humans who fail to satisfy them.”(Horta 2014, 142) For further discussion see: (Singer 2001; Horta 2014; Pluhar 2006; Dombrowski 2006; Kaufman 1998).

11 While I will not expand on the subject of potentiality, I must say that potentiality accounts generally have been found wanting. For a recent discussion see Stier and Schoene-Seifert (2013).

12 It must be clear that even if nobody’s dignity is denied in this case there can be other welfare considerations that should be taken into account when making a full ethical assessment of the creation of HNH-chimeras. For example, a chimerisation process, that is not identity affecting, could ‘confer’ dignity to a certain creature while at the same time it could impose serious bodily harms.

13 Even when presented in Johnston and Eliot’s paper as a “post-creation wrongful treatment argument”, this argument can also be interpreted as a “wrongful creation argument”.

14 In order for this counterargument to work we need to differentiate between two types of creation acts: ‘true’ creation acts (e.g. the fusion of two non-monozygotic early embryos) and creation as modification acts (e.g. grafting liver human cells into a post natal mouse). ‘True’ creation acts are characterised by the fact that without their occurrence the created entities would not have existed (i.e. the numerical identity of the creature depends on chimerisation act).
